# What influences the intention to get vaccinated against COVID-19 in the Western Balkans? Smiljana Cvjetkovic

**DOI:** 10.1093/eurpub/ckac131.367

**Published:** 2022-10-25

**Authors:** V Jeremic Stojkovic, S Cvjetkovic, A Stevanovic, J Jankovic, S Matovic-Miljanovic

**Affiliations:** 1 Department of Humanities, Faculty of Medicine of University of Belgrade, Belgrade, Serbia; 2 Institute of Social Medicine, Faculty of Medicine of University of Belgrade, Belgrade, Serbia; 3 Euro Health Group, Copenhagen, Denmark

## Abstract

**Background:**

Although effective vaccines against COVID-19 have been developed with the unprecedented speed, insufficient vaccine uptake suggests that many people are unwilling to get vaccinated worldwide. The aim of this study was to assess factors influencing intention to get vaccinated against COVID-19 in five Western Balkans countries.

**Methods:**

Total of 700 unvaccinated respondents aged 18-75 participated in the study. Intention was assessed by a single item gauging the likelihood of getting vaccinated on a 5-points Likert scale. Multiple linear regression was used to determine whether socio-demographics (gender, age, religiousness, educational level, employment status, presence of chronic diseases) and attitudes towards vaccination predict intention to get vaccinated.

**Results:**

Proportion of respondents willing to get vaccinated against COVID-19 ranged from 22.6% in Serbia to 40.4% in Montenegro. In Bosnia and Herzegovina and Albania stronger intention to get vaccinated in the future was associated with confidence in vaccine safety (β=.24, p<.01 and β=.20, p<.01 respectively) and efficacy (β=.28, p<.01 and β=.26, p<.001), higher feeling of danger of the disease (β=.21, p<.05 and β=.17, p<.05) and higher social responsibility (β=.35, p<.001 and β=.18, p<.01). Confidence in vaccine efficacy (β=.34, p<.001) and social responsibility (β=.24, p<.01), accompanied with higher sense of susceptibility to the disease (β=.13, p<.05), were significant predictors of intention in Serbia. In North Macedonia willingness to get the vaccine was significantly associated only with social responsibility (β=.09, p<.001), while in Montenegro age was the single predictor (β=-.32, p<.001);

**Conclusions:**

Results of this study suggest that vaccination campaigns should focus on specific set of socio-psychological factors in each country, enhancing confidence in vaccine efficacy and appealing to collective responsibility as most prevalent determinants of vaccination intention in Western Balkans.

**Key messages:**

• Specific set of socio-psychological factors influenced vaccination intention in each country.

• Most prevalent factors influencing intention to get vaccinated against COVID-19 were confidence in vaccine efficacy and social responsibility.

